# Authenticating the geographic origins of *Atractylodes lancea* rhizome chemotypes in China through metabolite marker identification

**DOI:** 10.3389/fpls.2023.1237800

**Published:** 2023-09-28

**Authors:** Chengcai Zhang, Hongyang Wang, Chaogeng Lyu, Yiheng Wang, Jiahui Sun, Yan Zhang, Zengxu Xiang, Xiuzhi Guo, Yuefeng Wang, Ming Qin, Sheng Wang, Lanping Guo

**Affiliations:** ^1^ State Key Laboratory for Quality Ensurance and Sustainable Use of Dao-di Herbs, National Resource Center for Chinese Materia Medica, China Academy of Chinese Medical Sciences, Beijing, China; ^2^ College of Horticulture of Nanjing Agricultural University, Nanjing, China; ^3^ Dexing Research and Training Center of Chinese Medical Sciences, China Academy of Chinese Medical Science, Dexing, China

**Keywords:** *Atractylodes lancea*, GC-MS, chemotype, VOC, metabolomic profiling, chemical markers, geographic origins

## Abstract

**Introduction:**

*Atractylodes lancea* is widely distributed in East Asia, ranging from Amur to south-central China. The rhizome of *A. lancea* is commonly used in traditional Chinese medicine, however, the quality of products varies across different regions with different geochemical characteristics.

**Method:**

This study aimed to identify the chemotypes of *A. lancea* from different areas and screen for chemical markers by quantifying volatile organic compounds (VOCs) using a targeted metabolomics approach based on GC–MS/MS.

**Results:**

The *A. lancea* distributed in Hubei, Anhui, Shaanxi, and a region west of Henan province was classified as the Hubei Chemotype (HBA). HBA is characterized by high content of β-eudesmol and hinesol with lower levels of atractylodin and atractylon. In contrast, the Maoshan Chemotype (MA) from Jiangsu, Shandong, Shanxi, Hebei, Inner Mongolia, and other northern regions, exhibited high levels of atractylodin and atractylon. A total of 15 categories of VOCs metabolites were detected and identified, revealing significant differences in the profiles of terpenoid, heterocyclic compound, ester, and ketone among different areas. Multivariate statistics indicated that 6 compounds and 455 metabolites could serve as candidate markers for differentiating *A. lancea* obtained from the southern, northern, and Maoshan areas.

**Discussion:**

This comprehensive analysis provides a chemical fingerprint of selected *A. lancea*. Our results highlight the potential of metabolite profiling combined with chemometrics for authenticating the geographical origin of *A. lancea*.

## 1 Introduction

*Atractylodes lancea*, known as “Cangzhu” in China, is a perennial herb belonging to the *Asteraceae* family. The rhizome of *A. lancea* (ALR) has been traditionally used in Chinese medicine (TCM) for its significant curative effects, which include drying dampness and strengthening the spleen, dispelling wind and cold, and improving eyesight (Zhang B. X. et al., 2020; Xu et al., 2022). Numerous pharmacological experiments, both *in vivo* and *in vitro*, have demonstrated that ALR exhibits liver protection, lowers blood glucose, has diuretic properties, and anti-hypoxic effects, as it contains various sesquiterpenoids and other medicinal components (Qin et al., 2019; Acharya et al., 2021; Zhang A. et al., 2021). One prominent sesquiterpenoid found in ALR is atractylodin (C_13_H_10_O). According to the Chinese Pharmacopoeia (Commission, 2020), the content of atractylodin (C_13_H_10_O) in ALR should be at least 0.30%. Atractylodin has been demonstrated to have anti-tumor, anti-bacterial, anti-viral, anti-arrhythmia effects, particularly on the nervous system and alimentary canal. Thus, it has been considered as one of the major constituents of ALR (Chae et al., 2016; Ma et al., 2019; Chang et al., 2021). Sesquiterpene compounds are the major bioactive agents in ALR essential oil. Among them, the three major components (hinesol, atractylon, β-eudesmol) have been identified to possess anti-sudorific, anti-bacterial, anti-tumor immunity, and blood glucose-lowering activities (Yuan et al., 2019; Wang et al., 2022a). For example, using the MTT assay, Guo et al. found that hinesol inhibited the proliferation of A549 and NCI-H1299 cells in a dose- and time-dependent manner; accordingly, hinesol is a potential drug candidate for the treatment of NSCLC (Guo W. et al., 2019). Similarly, β-eudesmol exerts anti-tumor and anti-angiogenic activity and can act as a chemosensitizing agent in therapies targeting drug-resistant cancers (Acharya et al., 2021). Cheng et al. (2022) showed that atractylon regulates the expression of two cancer-associated RNA molecules, TMPO-AS1 and CCDC183-AS1, and inhibits the invasion and migration of liver cancer cells. They also showed that atractylon has anti-hypertensive, anti-aging, and anti-inflammatory effects (Cheng et al., 2022). Previous studies have demonstrated that the high-quality *A. lancea* that is produced in the Maoshan area is enriched in both atractylodin and atractylon (Wang et al., 2012; Xu et al., 2022). In fact, Wu et al. (2023) showed that *A. lancea* can be divided into two chemotypes, including the Maoshan chemotype as well as a chemotype associated with the Dabieshan region. Gas chromatography-mass spectrometry (GC-MS) analyses revealed that the levels of hinesol and β-eudesmol in the Dabieshan chemotype were higher than those in the Maoshan chemotype (Wu et al., 2023). Together, these four essential oils (β-eudesmol, hinesol, atractylodin, and atractylon) thus serve as indicator components for evaluating the quality of *A. lancea*, and we therefore selected them as critical compounds for characterizing the chemotypes.

The distribution and quality of *A. lancea* are influenced by climate variations between different geographic regions. The main production areas, Jiangsu, Hubei, and Anhui, have temperate or subtropical climates characterized by aridity and minimal rainfall in spring, along with high temperatures in summer. Previous studies have revealed that high temperature and humidity, a period of intense heat and rainfall, and low‐potassium stress contribute to the formation of high-quality *A. lancea* in the Jiangsu Maoshan area (Wang et al., 2022a; Wang et al., 2022b). Geographic disparities in growing areas result in variations in the quality of ALRs, with the drug sourced from Jiangsu Province demonstrating the best clinical efficacy. For instance, a variaty of *A. lancea* known as Maoshan Cangzhu (Maocangzhu in Chinese), native to Maoshan of Jiangsu Province, China, exhibits higher quality due to its elevated essential oil content. Moreover, the cross section of the Maocangzhu rhizome exhibits brown-red oil glands, which are regarded as desirable traits contributing to its superior quality (Hao and Xiao, 2018; Jiang et al., 2022).

Recent studies have demonstrated that ALR extract inhibits tumor cell proliferation without damaging normal cells, especially in treatment of gastrointestinal cancers (Alhosseini et al., 2021; Guo S. et al., 2019). Additionally, *A. lancea* has played a significant role in combating COVID-19 in China and ranks as the primary TCM for national epidemic prevention and control. Concequently, the demand for *A. lancea* is rapidly increasing, and high-quality *A. lancea* may have even more medicinal uses. However, the quality of ALR from different habitats varies, and the specific compounds responsible for the variation in ALR from different origins remain unclear. Furthermore, there is limited direct evidence regarding the variation in the concentration of these volatile organic compounds (VOCs), especially terpenoids, heterocyclic compounds, esters, and ketones in *A. lancea* from different geographical locations.

Plant VOCs encompass organic compounds with boiling points ranging from 50 to 260°C under normal pressure. These compounds include terpenoids, aromatic compounds, esters, hydrocarbons, and aldehydes (Wang et al., 2007). VOCs can act as information-delivering chemicals in multitrophic interactions between plants and their environment, including interactions with other plants, which holds great significance for environmental security and human survival (Yuan et al., 2009; Dudareva et al., 2013). The VOCs derived from ALR not only have significant pharmacological effects, but also play an important role in the plant adaptation to the environment.

We propose that the variations in the quality of ALRs grown in different habitats are ultimately reflected in the terminal metabolites of the metabolic pathway, Additionally, specific chemotypes of *A. lancea* may develop in response to environmental factors. Secondary metabolites in medicinal plants can be gradually generated in response to environmental stress (Yang et al., 2018), and numerous studies have shown that production environment has a great influence on the quality of herbs from different distribution areas (Sun et al., 2020; Zhang et al., 2022). The importance of authenticating and tracing the geographical origin of *A. lancea* has promoted investigations of the chemical diversity of the specialized metabolites. Metabolomics, a comprehensive and global analysis of diverse metabolites produced in cells and organisms, offers an accurate means of assessing the quality differences of Chinese medicinal materials (CMMs) resulting from environmental, planting, or genetic factors (Zhang et al., 2018; Kang et al., 2020; Lyu et al., 2022). However, to date, there is no published comprehensive and systematic study focusing on the identification of chemotypes and profiling analyses of VOC metabolites from ALR sourced from different geographical areas. Thus, a detailed profile of metabolites in ALR is important for comprehensively evaluating its medicinal value, elucidating the compounds underlying the observed variation and understanding its related efficacy.

The objectives of this study were as follows: (i) to comprehensively evaluate the metabolic profile of *A. lancea*, (ii) to identify candidate chemical markers for distinguishing *A. lancea* samples collected from different distribution areas, (iii) to determine the chemotypes of *A. lancea* from various geographical areas and (iv) to establish the correlation between origin classification and major chemical markers. In the present research, GC-MS, hierarchical clustering analysis (HCA), principal component analysis (PCA) and supervised partial least square discriminant analysis (PLS-DA) were employed to study the differences of metabolites and screen for biomarkers in ALR from different distribution areas. Our work elucidates the variation characteristics of *A. lancea* essential oil and provides valuable data for geographical origin identification, quality assessment, and standardized cultivation of *A. lancea.*

## 2 Materials and methods

### 2.1 Plant materials

The rhizomes of wild *A. lancea* were collected in the summer (2-20 July, 2019-2021) from 13 different distribution areas in the provinces of Shanxi, Inner Mongolia, Hebei, Jiangsu, Hubei, Anhui, Shandong, Henan and Shaanxi in China. Specifically, these samples were collected from Wuzhai, Shanxi; Zhalantun, Neimenggu; Longhua, Hebei; Chengde, Hebei; Jurong, Jiangsu; Jiangning, Jiangsu; Yunxian, Hubei; Lu’an, Anhui; Kunyushan, Shandong; Huoshan, Anhui; Luotian, Hubei; Songxian, Henan; and Xunyi, Shaanxi; etc. One hundred thirty-three *A. lancea* samples from 13 areas in China were ultimately isolated. The collected wild *Atractylodes lancea* were identified by researcher Guo Lanping from the National Resource Center for Chinese Materia Medica, China Academy of Chinese Medical Sciences as the dry rhizome of *Atractylodes lancea* belonging to the *Asteraceae* family. In addition, their ages, which appear to be more than 3 years old, differ among the individual plants. The morphological differences of rhizomes samples collected were shown in **Figures S1**, **S2**.

The rhizomes were washed with distilled water, dried in the shade, sealed in small polyethylene plastic bags, and stored in a refrigerator (FYL-YS-310L, Beijing, China) at 4°C. Detailed sample information is displayed in **Supplementary Table 1**. Chemical standards (>98% purity), including atractylodin, hinesol, atractylone, and β-eduesmol, were purchased from Shanghai Yuanye Bio-Technology Co., Ltd (Shanghai, China), and standards were accurately weighed and dissolved in hexane at a concentration of approximately 1 mg/mL.

### 2.2 Quantitative analysis of ALR essential oils

The essential oil contents of ALRs from 13 distribution areas were determined by GC-MS analyses. The ALR samples were first ground into powder, then filtered through a sieve with screen mesh size of 60 (sieve size No. 3, as defined by the Chinese Pharmacopoeia (2020) (Jun et al., 2018)). Approximately 500 mg of each ALR sample was placed into a 50 mL centrifuge tube followed by the addition of 20 mL hexane. The clear supernatant fluid was decanted after ultrasonic processing (40 kHz, 30 min) and centrifugation (3000 rpm for 10 min at room temperature). The drug residue was extracted with an additional 20 mL hexane following the same procedure. The volume of the combined supernatants was adjusted to 50 mL with hexane, and the mixture was filtered.

A portion (1 μL) of the sample was injected into a GC system (ThermoFisher, Waltham, MA, USA) that was coupled to a triple quadrupole MS (TP-8030). The GC was fitted with a DB-5MS capillary column (0.25 mm × 30 m, 0.25 μm particle size). Helium was the carrier gas with a 1 mL/min flow rate. The injection mode used the split-flow technique (split ratio, 50:1). The column temperature was 120°C for the first 2 min, and then increased to 240°C at 5°C/min and held for 5 min. The GC–MS was operated in electron ionization (EI) mode at 70 eV, with an ion source temperature of 230°C and a quadrupole mass spectrometer temperature of 150°C, and the MS scanned from m/z 40-500 in the MSD data acquisition mode.

### 2.3 Data analysis

Quantitative experimental data are presented as the mean ± standard deviation of three independent experiments. One-way analysis of variance (ANOVA) was applied using IBM SPSS Statistics version 26 (SPSS, Chicago) to assess the differences between mean values, followed by Duncan’s multiple comparisons with a 95% confidence level. HCA was employed to investigate similarities and relationships among plants from different distribution areas and was performed with SPSS software. Average linkage within groups was used for clustering, and Squared Euclidean Distance was selected.

### 2.4 Sample preparation for VOC metabolomic analysis

*A. lancea* rhizome samples were harvested, weighed, immediately frozen in liquid nitrogen, and stored at -80°C until use (we think that the sample was fresh without special instructions). Samples were ground to a powder in liquid nitrogen. Approximately 500 mg (1 mL) of the powder was transferred immediately to a 20 mL headspace vial (Agilent, Palo Alto, CA, USA), containing a saturated NaCl solution to inhibit enzyme-catalyzed reactions. The vials were sealed using crimp-top caps with TFE-silicone headspace septa (Agilent). At the time of SPME analysis, each vial was incubated at 60°C for 5 min, then a 120 μm DVB/CWR/PDMS fiber (Agilent) was exposed to the headspace of the sample for 15 min at 100°C.

### 2.5 GC-MS/MS conditions for VOC metabolomic analysis

After sampling, desorption of the VOCs from the fiber coating was carried out in the injection port of the GC apparatus (Model 8890; Agilent) at 250°C for 5 min in the splitless mode. The identification and quantification of VOCs was carried out using an Agilent Model 8890 GC and a 7000D MS (Agilent), equipped with a 30 m × 0.25 mm × 0.25 μm 5% phenyl-polymethyl siloxane (DB-5MS) capillary column. Helium was used as the carrier gas at a linear velocity of 1.2 mL/min. The injector temperature was maintained at 250°C, and the oven temperature program was as follows: 40°C for 3.5 min, increase at 10°C/min to 100°C, increase at 7°C/min to 180°C, increase at 25°C/min to 280°C, hold for 5 min. Mass spectra were recorded in electron impact (EI) ionization mode at 70 eV. The quadrupole mass detector, ion source and transfer line temperatures were 150, 230 and 280°C, respectively. The MS was run in selected ion monitoring (SIM) mode for the identification and quantification of analytes.

### 2.6 Multivariate statistical analysis

Qualitative and quantitative analyses of mass spectral data were performed with MassHunter software, and total ion currents (TIC) of the QC samples are shown in **Figure S3** (Zhang C. et al., 2020). Quantitative analyses were executed for each VOC in the ALR samples by extraction of a single characteristic ion. Relative quantifications of identified compounds in samples to be compared were based on the mean ionic intensity of the characteristic ions (Acharya et al., 2021; Xu et al., 2022). VOC metabolite data from 133 of *A. lancea* samples were used for PCA, HCA, and PLS-DA using Metware Clond, a free online platform for data analysis (https://cloud.metware.cn).

The obtained PCA model was verified using the QC sample. Heatmaps were created in MetaboAnalyst 5.0 to visualize the variations of differential VOC metabolites. The differentially accumulated VOC metabolites (DVMs) were identified based on the fold-change (Log_2_FC ≥ 2 or ≤ 0.5) and variable importance in project scores (VIP ≥ 1), which were extracted from PLS-DA results. Mean decrease in accuracy (MDA) values measure the contribution of individual metabolites in classification of the *A. lancea* samples from different distribution areas when using random forest (RF) algorithms. Metabolites with MDA values > 0.004 were considered to play an important role in discriminating the *A. lancea* from different regions. Heatmaps were generated for metabolites with a combination of VIP scores ≥ 1.0 and MDA > 0.004. The heatmaps were generated using Euclidean distance measuring and the Ward method as a clustering algorithm.

## 3 Results

### 3.1 Determination of essential oil content in *A. lancea* from different distribution areas

In this study, 133 dried ALR samples obtained from 13 different distribution areas were collected to investigate differences of main essential oil components (**Table 1**). We considered that sesquiterpenoids (β-eudesmol, hinesol and atractylon) and polyacetylene (atractylodin) are the major active components in *A. lancea*, and we focused on quantifying these four essential oil components in plants from different distribution areas. As shown in **Table 2**; **Supplementary Table 2**, the contents, and compositions of essential oils in *A. lancea* from different distribution areas were clearly different.

**Table 1:** Sample number information of *Atractylodes lancea* Rhizome from distribution areas.

Region	Longitude	Latitude	Altitude/m	Niche habitat	Number of samples
HBYX	110.753244	33.015596	839.49	Daliu Township, Yun County, Shiyan City, Hubei Province	11
JSNJ	119.017283	32.061882	121.60	Tangshan town, Jiangning District, Nanjing City, Jiangsu Province	13
HBLT	115.737272	31.080283	663.30	Tiantangzhai, Luotian County, Huanggang City, Hubei Province	6
SXWZ	111.4926	38.5548	1367.50	Zhongsuo village, Wuzhai County, Xinzhou City, Shanxi Province	8
AHHS	115.974057	31.086172	406.70	Shangtu Town, Huoshan County, Lu’an City, Anhui Province	9
AHLA	116.224063	31.688302	64.30	Shizigang Township, Yu’an District, Lu’an City, Anhui Province	10
SDKYS	121.771111	37.225556	250.00	Wuran Temple nature reserve, Kunyu Mountain, Yantai City, Shandong Province	3
NMGZLT	122.264444	47.534444	380.00	Sama Street Ewenki nationality township, Zalantun City, Hulunbuir City, Inner Mongolia Autonomous Region	9
JSJR	119.125278	32.066944	244.00	Huangmei Town, Jurong City, Zhenjiang City, Jiangsu Province	13
HBCD	117.940556	40.888056	340.00	Feng yingzi Zhen Feng yingzi Cun, Shuangqiao District, Chengde City, Hebei Province	15
HBLH	117.91	41.3575	700.00	Wafang village, zhangjiying Township, Longhua County, Chengde City, Hebei Province	10
HNSX	112.14538	33.750052	1158.19	Che Cun Nan Shan, Song County, Luoyang City, Henan Province	11
SXXY	108.595942	35.060763	1623.45	Miao Gou Po Shan, Xunyi County, Xianyang City, Shanxi Province	15

**Table 2:** Comparison of the levels of four *A. lancea* essential oils according to geographical area.

Region	Atractylodin (mg/g)	Atractylon (mg/g)	Hinesol (mg/g)	β-eudesmol (mg/g)	Component proportion
SXWZ	0.74 ± 0.63efg	0.30 ± 0.06g	1.25 ± 1.73d	1.94 ± 2.75cd	0.38:0.15:0.64:1
NMGZLT	3.49 ± 1.71a	2.21 ± 1.41ab	1.08 ± 1.15d	1.56 ± 1.86cd	2.24:1.42:0.69:1
HBLH	2.36 ± 1.06abcd	1.52 ± 1.24bcde	0.75 ± 0.97d	1.18 ± 1.53cd	2:1.29:0.64:1
HBCD	1.54 ± 0.86defg	1.39 ± 1.17bcdef	0.29 ± 0.49d	0.39 ± 0.78d	3.95:3.56:0.42:1
JSJR	2.88 ± 1.07abc	3.12 ± 1.30a	0.23 ± 0.34d	0.33 ± 0.52d	8.73:9.45:0.69:1
JSNJ	1.78 ± 0.66cde	1.94 ± 1.04abcd	0.42 ± 0.45d	0.65 ± 0.72d	2.74:2.98:0.65:1
HBYX	0.40 ± 0.47g	0.77 ± 0.62defg	17.57 ± 11.81a	6.58 ± 3.39ab	0.06:0.12:2.67:1
AHLA	1.73 ± 0.76cdef	2.06 ± 1.54abc	7.83 ± 4.84c	7.87 ± 2.50a	0.22:0.26:0.99:1
SDKYS	3.29 ± 2.42ab	0.27 ± 0.25fg	0.11 ± 0.16d	0.07 ± 0.09d	47:3.86:1.57:1
AHHS	0.42 ± 0.77fg	0.88 ± 0.69cdefg	13.52 ± 6.24ab	7.13 ± 4.04a	0.06:0.12:1.90:1
HBLT	0.46 ± 0.45fg	1.06 ± 0.61bcdefg	12.00 ± 2.46abc	7.72 ± 4.19a	0.06:0.14:1.55:1
HNSX	0.76 ± 1.51efg	0.30 ± 0.53efg	9.79 ± 3.94bc	3.80 ± 3.03bc	0.2:0.08:2.58:1
SXXY	2.15 ± 1.32bcd	0.84 ± 0.73cdefg	8.85 ± 4.54bc	7.71 ± 3.50a	0.28:0.11:1.15:1
Total	1.68 ± 1.40	1.37 ± 1.29	5.61 ± 7.30	3.60 ± 3.97	0.47:0.38:1.56:1

Different letters in the same column indicate significant differences between plants (p < 0.05).

**Figure 1:**
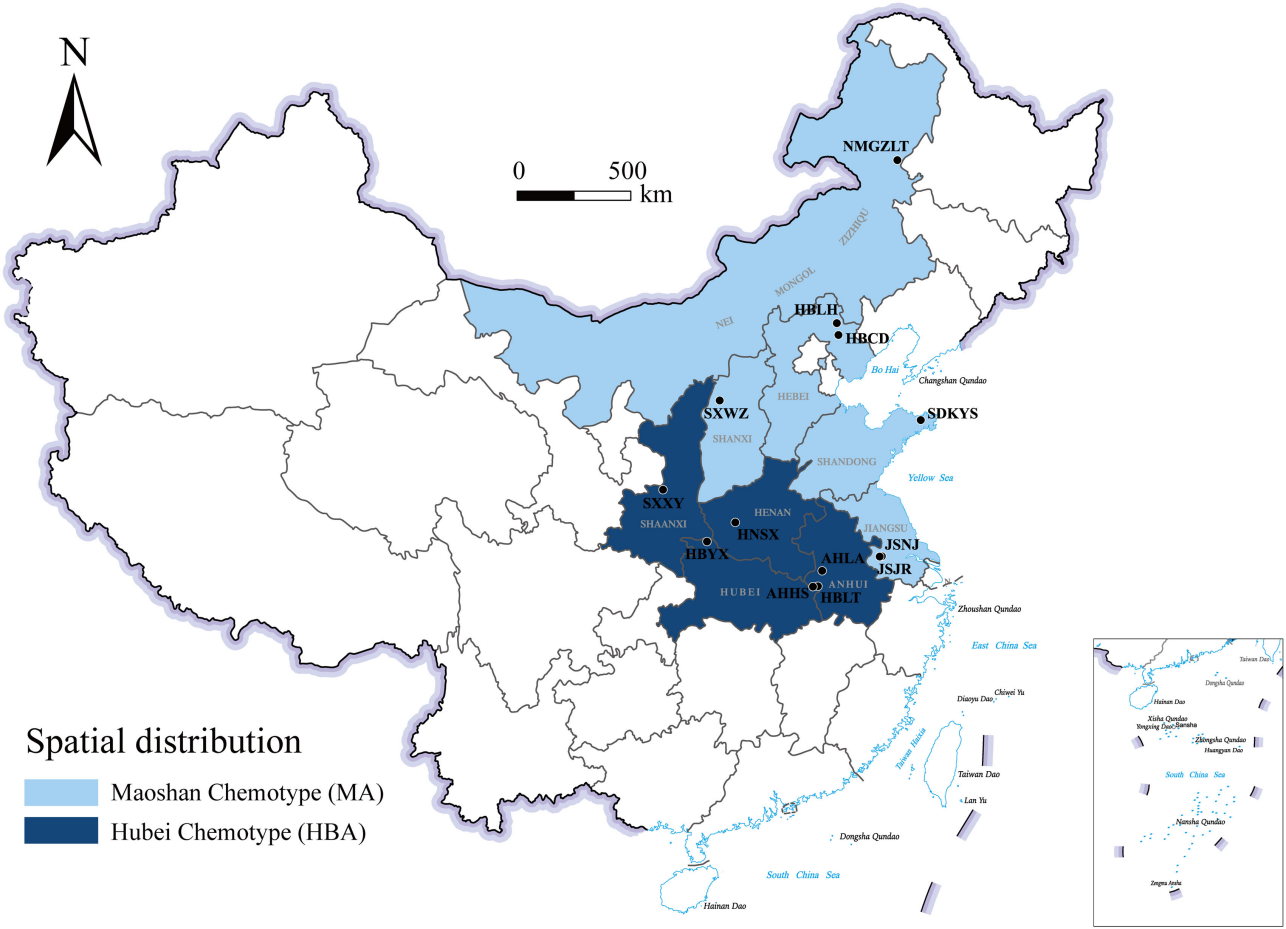
Geographical distribution of the main *A. lancea* chemotype clusters. JSJR, Jiangsu-Jurong; JSNJ, Jiangsu-Nanjing; SXWZ, Shanxi-Wuzhai; NMGZLT, Neimenggu-Zhalantun; HBCD, Hebei-Chengde; HBLH, Hebei-Longhua; HBYX, Hubei-Yunxian; AHLA, Anhui-Lu’an; AHHS, Anhui-Huoshan; HBLT, Hubei-Luotian; HNSX, Henan-Songxian; SXXY, Shaanxi-Xunyi; SDKYS, Shandong-Kunyushan.

A comparison of these results led us to identify two chemotypes of *A. lancea* essential oil in China. One chemotype, called the Hubei chemotype (HBA), has hinesol and β-eudesmol as the main components with atractylodin and atractylon as minor components. Plants with this chemotype were mainly distributed in the south-central region of China, especially in the provinces of Hubei, Anhui, and Shaanxi, as well as a region west of Henan (**Figure 1**). In addition, ALRs of this chemotype were also characterized by a higher total content the four measured VOCs.

The other chemotype was characterized by higher levels of atractylodin and atractylon and was called the Maoshan chemotype (MA). Plants with this chemotype were distributed throughout the provinces of Hebei and Inner Mongolia, in the northern region of Shandong and in the southern region of Jiangsu (**Figure 1**). Interestingly, we found that the chemical constitution of essential oils from *A. lancea* in SXWZ was distinctive, as extracts from these plants had higher levels of hinesol and β-eudesmol but relatively low total essential oil contents.

**Figure 2:**
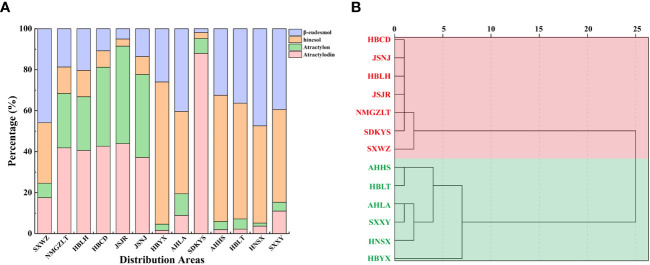
Comparison of essential oils from *A. lancea* growing in different regions. **(A)** Composition of essential oils in *A. lancea* from 13 distribution areas. **(B)** Dendrogram showing the levels of four main components of essential oils from *A. lancea*. Hierarchical clustering was performed with SPSS software. Average linkage within groups was used for clustering, and Squared Euclidean Distance was selected.

Taken together, these results revealed geographic variations in the total amount of essential oil and the levels of four key VOC components in *A. lancea*. The *A. lancea* produced in the geo-herb area (JSNJ and JSJR) belongs to the MA chemotype, and the average atractylodin: atractylon: hinesol:β-eudesmol ratios were (2.74:2.98:0.65:1), (8.73:9.45:0.69:1) in *A. lancea* from JSNJ and JSJR (geo-herb production area), respectively (**Table 2**; **Figure 2A**).

Upon the performing of cluster analyses of *A. lancea* populations based on the four main essential oil components, we identified two main clusters that incorporated the 13 A*. lancea* populations (**Figure 2B**). Populations from AHHS, HBLT, HBYX, HNSX, SXXY, as well as a sub-population from AHLA, exhibited a similar cluster, which was designated HBA. *A. lancea* populations from NMGZLT, HBCD, HBLH, an area of SDKYS, and the Jurong, Nanjing region of Jiangsu province (JSJR, JSNJ) were clustered into a second group, designated MA. Among these, the chemotypes of plants from NMGZLT and SXWZ are similar. These results of cluster analysis considering the four main compounds demonstrated that the essential oil composition in plants from MA was significantly different from that of plants from HBA. The content of four main essential oil components varies across different regions, and the composition exhibits a certain correlation with geographical distribution.

### 3.2 Profiles of VOCs and their concentrations in *A. lancea* extracts

GC-MS/MS analyses were employed to develop comprehensive profiles of the VOCs from ALR from *A. lancea* from 13 regions of origin. The metabolic profiles can be observed as chromatographic peaks in the TIC (**Supplementary Figure 2**); a high ratio of overlap of the TIC curves was found for the various samples, implying that the results were repeatable and reliable.

**Figure 3:**
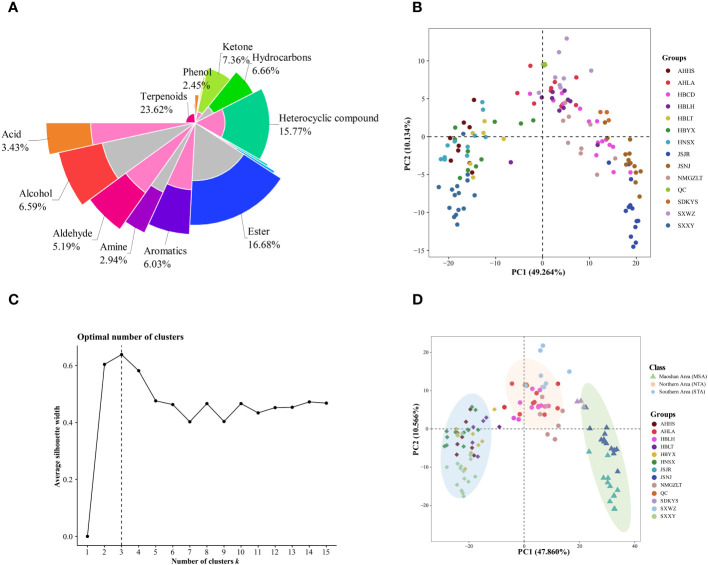
Multivariate statistical analyses of *A. lancea* from different distribution areas based on GC-MS/MS profiles. **(A)** Nightingale Rose Chart representing the proportions of volatile metabolites in all *A. lancea* rhizome (ALR) samples. **(B)** Score plot of PCA-based VOC profiling of ALR from 133 distribution areas. **(C)** Averaged clustering balance of all possible numbers of clusters using a *k*-means analysis. **(D)** Optimal three group classification based on volatile metabolome data from 114 production areas.

In this series of experiments (**Supplementary Table 3**), 1427 compounds were detected, including 337 terpenoids, 238 esters, 225 heterocyclic compounds, 105 ketones, 95 hydrocarbons, 94 alcohols, 86 aromatic compounds, 74 aldehydes, 49 acids, 42 amines, 35 phenols, 17 sulfur compounds, 11 nitrogen compounds, 7 ethers, 6 halogenated hydrocarbons, and 6 additional compounds that did not fit into these 15 main classes. These metabolites and their relative abundances are shown graphically as different colored blocks in **Figure 3A**. Among these metabolites, the largest group was the terpenoids, the relative content of which accounted for 23.62% of the total metabolite composition, followed by esters, heterocyclic compounds, and ketones.

Differences in the VOCs content of ALRs from different distribution areas were discriminated using an unsupervised PCA model. As shown in **Figure 3B**, the QC samples formed clear clusters, and their positions were near the co-ordinate origin, which confirmed the stability of the analytical method. In addition, the location of origin of each *A. lancea* sample could be roughly distinguished. In the PCA score plot, two principal components, PC1 and PC2, were extracted, and these components accounted for 49.264% and 10.134%, respectively, of the total variance, meaning that the cumulative contribution of these principal components was 59.398%.

The wide range of *A. lancea* samples tested may have led to a relative lack of clarity of separation. Therefore, a method for determining the optimal number of clusters based on an agglomerative hierarchical clustering algorithm was conducted. From the data displayed in **Figure 3C**, it is clear that the highest silhouette score, at *K* = 3, indicates that the optimum number of clusters is 3, and the original PCA classification results based on this *k*-means analysis is shown in **Figure S4**.

In order to better classify the metabolic profiles of samples from different regions, we excluded four sampling sites (NMGZLT-4, NMGZLT-5, HBLH-1 and HBLH-10) that exhibited relatively highly fluctuating data; these data may have been subjected to experimental error or to the mixing of samples. The HBCD sample site was also excluded due to a lack of clarity among the groups (**Supplementary Table 4**). The differences in VOCs profiles among samples from 114 distribution areas can be seen in the optimized PCA plot representing metabolite groups (**Figure 3D**). Here, 12 sampling locations were found to form three distinct groups (MSA, NTA and STA), in agreement with the results of the *k*-means analysis.

**Figure 4:**
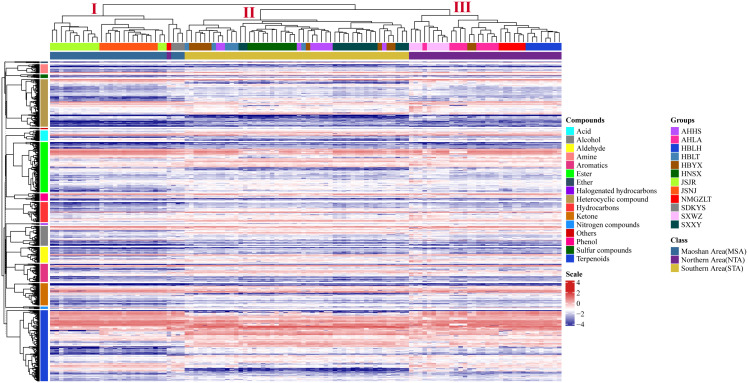
Hierarchical clustering heatmap of compounds from all samples of *A. lancea*. A heatmap showing the relative variations in the volatile metabolite profiles of *A. lancea* from different distribution areas. Each sample from the noted distribution areas is represented in one column, and each metabolite is represented by a raw element.

The differences in VOC concentrations among samples from different distribution areas can be seen in the heatmaps of metabolite content (**Figure 4**; **Supplementary Table 5**). HCA according to the Euclidean distance suggested that all samples can be divided to three clusters (I, II, and III) on the abscissa axis, which was consistent with the results of PCA. Cluster I was further divided into three subclusters where the first subcluster (I) includes JSJR, JSNJ, and SDKYS; the second subcluster (II) consists of HBLT, HBYX, AHHS, HNSX, and SXXY; and the third subcluster (III) consists of SXWZ, AHLA, NMGZLT, and HBLH. Thus, together, PCA and HCA analyses suggested that the samples of the MSA, NTA, and STA regions had clearly distinct metabolic profiles.

### 3.3 Metabolomics analysis of samples of *A. lancea* from different distribution areas

**Figure 5:**
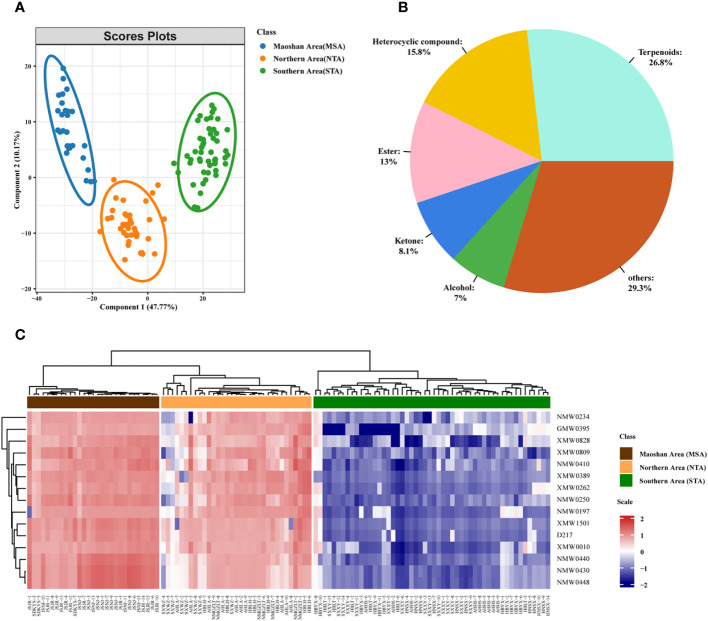
Metabolomic analyses of *A. lancea* according to geographic origin. **(A)** PLS-DA score plot of three groups of *A. lancea* based on the volatile metabolome data from 114 production areas. STA, Southern Area; NTA, Northern Area; MSA, Maoshan Area. **(B)** Classification and percentage of significantly different metabolites among plants from the three major regions of *A. lancea*. **(C)** Heatmap of 15 differential metabolites among MSA, NTA and STA.

A supervised PLS-DA was also performed on the VOC profiles. From the PLS-DA score plot (**Figure 5**), it can be seen that the three distinct groups (MSA, NTA and STA) were distinctly separated from each other. Cross-validation of this PLS-DA model led to correlations of R^2^X = 0.956 and R^2^Y = 0.939, and the cumulative Q^2^ value was 90.7% (**Figure S5**), indicating that the PLS-DA models can be relied upon to classify new data sets into groups.

We also calculated the ANOVA *p* value and VIP score of the VOCs based on the PLS-DA model. Using ANOVA *p* < 0.05 and VIP score ≥ 1 as a cutoff, a total of 455 differentially abundant metabolites were selected and determined to be candidate markers for the differentiation of *A. lancea* from different distribution areas. A complete list of these compounds is given in **Supplemental Table 6**. They belong to terpenoids (122 out of 445, 26.8%), heterocyclic compounds (72 out of 445, 15.8%), esters (59 out of 445, 13.0%), ketones (37 out of 445, 8.1%), alcohols (32 out of 445, 7.0%) and other compounds (**Figure 5B**; **Supplementary Table 6**). After further examination, the numbers were narrowed down to 15 (**Supplementary Table 7**; **Figure 5C**). Heatmaps that graphically represent variations in metabolite signal intensities for the three groups are presented in **Figure 5C**. As a result of these analyses, the level of VOCs in the MSA cluster was found to be slightly higher than that of NTA, but the difference was not significant. On the other hand, the level of VOCs in the MSA cluster was significantly higher than that in the STA cluster.

### 3.4 Exploratory analysis of the chemical markers in *A. lancea*

In contrast to PLS-DA and unsupervised hierarchical clustering, RF analysis is an unbiased and supervised classification technique based on an ensemble of a large number of decision trees. PLS-DA is prone to over-fitting and may select metabolites that are ultimately biologically irrelevant. In contrast, RF analysis is resistant to over-fitting when parameters are properly optimized. Accordingly, both PLS-DA and RF analysis were applied to identify DVMs among MSA, NTA and STA. Metabolites that met a combination of inclusion criteria, including PLS-DA VIP scores > 1.0 and RF mean decrease in accuracy (MDA) values > 0.004, were selected for the model.

**Figure 6:**
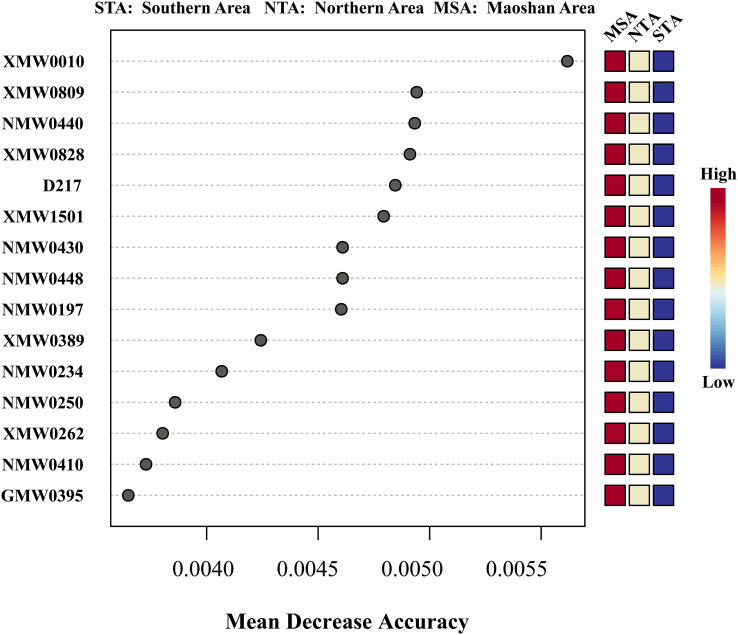
Random forest mean decreases in accuracy (MDA) scores for the top 15 differential metabolites for training data set among MSA, NTA and STA. Only metabolites with MDA > 0.004 were considered for inclusion in the final model. The colored column to the right of each figure displays the variations in individual metabolite peak intensities compared between groups. Red indicates an increase in peak intensity and blue is a decrease.

The top 15 MDA scores for metabolites from three major regions (MSA, NTA, and STA) are listed in **Figure 6**. Among the top 15 differential metabolites, ketobemidone; 2,2’-bis(4,5-dimethylimidazole); 1,3-pentanedione; 4,4-dimethyl-1-phenyl-(E, E, E)-2,6,9,9-tetramethyl-2,6,10-cycloundecatrien-1-one; β-santalol and cembrene were the most important and reliable chemical markers according to the RF exploration analysis (**Figure 6**), implying that they are optimally capable of discriminating NTA, STA and MSA.

### 3.5 Identification of *A. lancea* using the chemical markers

**Figure 7:**
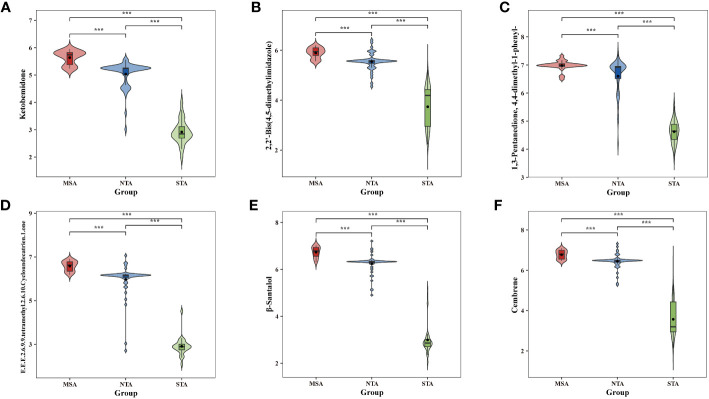
Comparative analysis of the six chemical markers metabolites among MSA, NTA and STA regions. **(A)**, Ketobemidone; **(B)**, 2,2'-Bis (4,5-dimethylimidazole); **(C)**, 1,3 Pentanedione, 4,4-dimethyl-1 phenyl-; **(D)**, (E,E,E)-2,6,9,9-tetramethyl-2,6,10-Cycloundecatrien- 1-one; **(E)**, β-Santalol; **(F)**: Cembrene.

The potential marker compounds in the three groups were selected using multivariate (PLS-DA), univariate (ANOVA), and RF analyses. Overall, six DVMs showed significant differences among the MSA, NTA and STA regions (**Supplementary Table 8**). Violin charts (**Figure 7**) show that the concentration of all chemical markers was significantly upregulated in the MSA group, followed by that of NTA and STA, whereas the content of cembrene and 2,2’-bis(4,5-dimethylimidazole) in MSA was slightly higher than that of NTA, but the difference was not significant. The results indicated that the six selected chemical markers can effectively distinguish A. lancea from different distribution areas.

### 3.6 Analysis of correlation between the chemical markers and four essential oils in different distribution areas

**Figure 8:**
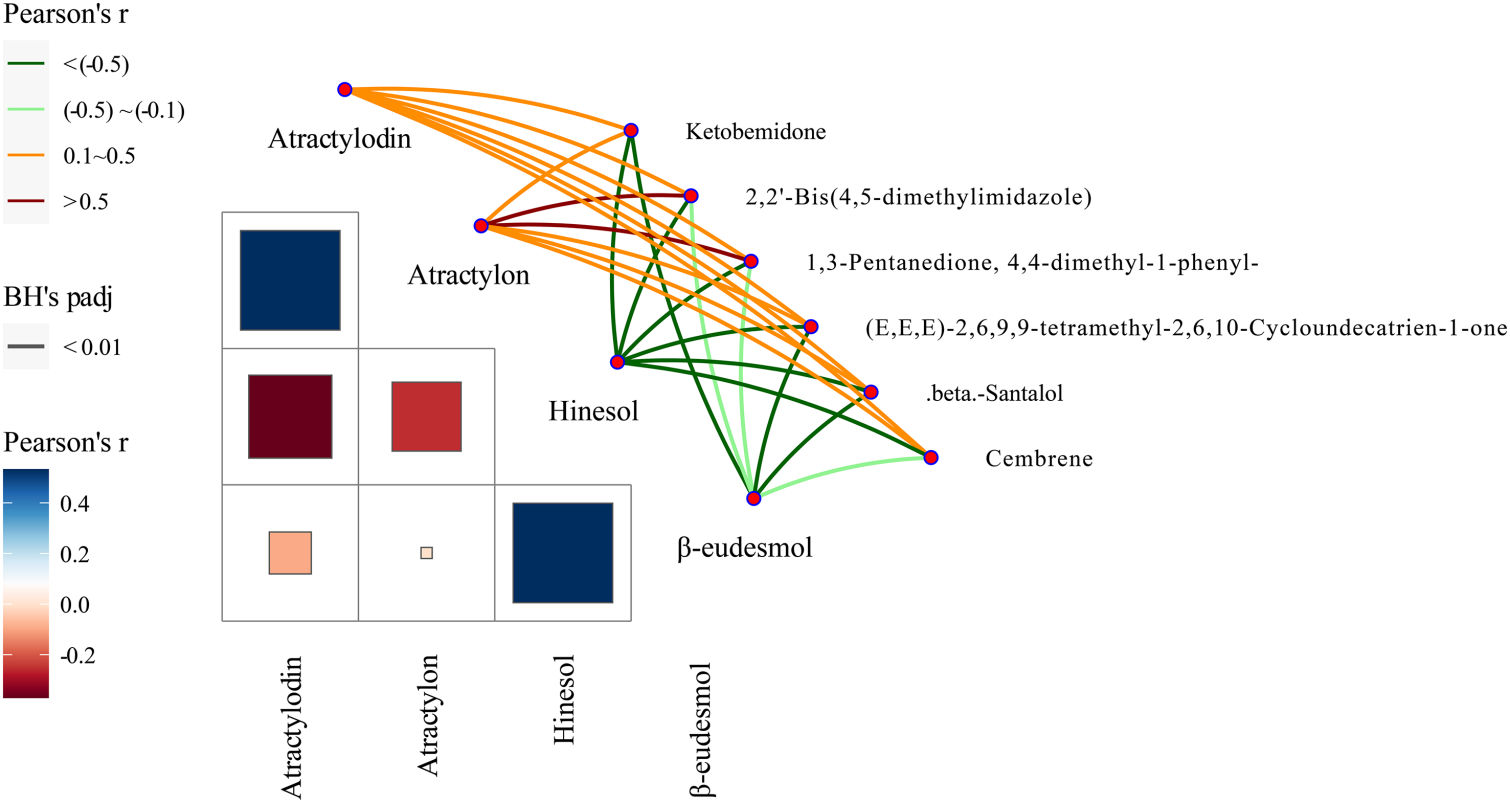
Correlations between levels of the six chemical markers and of the four essential oils according to the Mantel test. Pearson’s r represents the correlation coefficients among different compounds with a color gradient denoting Spearman’s correlation coefficient. Mantel’s r reveals the correlation coefficients between six chemical markers and four essential oils; thicker lines represent larger correlation coefficients. BH’s padj represents the significance of the correlation, and edge color denotes the statistical significance based on 9999 permutations.

Our previous studies suggested that the four *A. lancea* essential oil components studied here, atractylodin, β-eudesmol, hinesol, and atractylon, could be used as chemical markers for the authentication and traceability of the geographic origins of plant samples. The six metabolite markers were further verified by analysis of the four essential oils components using the Mantel test across 13 A*. lancea* populations. In the present study, the six chemical markers were found to positively correlate with both atractylodin and atractylon in the surface layer, while significant negative correlations were found for these markers with hinesol and β-eudesmol (**Figure 8**). Most notably, atractylon showed a high significant positive correlation with 2,2’-bis(4,5-dimethylimidazole) and 1,3-pentanedione, 4,4-dimethyl-1-phenyl. It is widely known that the ALR from the Dao-di area has higher contents of atractylodin and atractylon. These verification results showed that these chemical makers are useful for the identification of origin and quality assessment of *A. lancea*.

## 4 Discussion

*A. lancea* exhibits a wide distribution across various regions in China, including cold temperate zone, middle temperate zone, north subtropical region, and middle subtropics (Liu et al., 2016). Each of these regions provides a unique growing environment for *A. lancea*, characterized by distinct climatic conditions. For instance, Maoshan region offers a suitable environment with high humidity and warm temperatures, while Inner Mongolia provides a cold and dry climate that favors the growth of *A. lancea* (Zhou et al., 2018). The variations in habitats strongly influence the *A. lancea* germplasm, and consequently contribute to differences in the quality of *A. lancea* products (Tsusaka et al., 2019). These differences in quality ultimately manifest in the terminal metabolites of the metabolic pathway. Thus, this study aimed to develop metabolomics-based strategies for chemical type identification and to perform metabolite profiling analyses of ALR from different distribution areas.

### 4.1 Geographical variation and chemical type classification of *A. lancea* essential oil

*A. lancea* is a highly polymorphic species with continuous variation in the depth of the intra-species leaf split, the size of inflorescences and leaf shapes. Similarly, the volatile oil components of *A. lancea* showed continuous variation from south to north, allowing for research focused on species characterization and the identification of chemical types within species. Notably, significant contributions in this field have been made by Takeda and colleagues in Japan, who conducted comprehensive analyses of the geographical variation in the volatile oil of *A. lancea* and the division of chemical types within the species. Using gas chromatography (GC) analysis, they systematically investigated the volatile oil of wild *A. lancea* in China and classified the chemical types of *A. lancea* into three groups according to the six main components of volatile oil, namely hinesol, β-eudesmol, atractylon, atractylodin, elemol and atractylodin lactone. In an early study, *A. lancea* of Jiangsu, Hubei, Shaanxi, and southern Henan were divided into three chemical types: Dabie Mountain Cangzhu, Hubei-Anhui Cangzhu and Maoshan Cangzhu. Subsequently, *A. lancea* in Hebei, Shandong, northern Henan, and northern Jiangsu were divided into two chemical types, with the Yellow River as the boundary. Finally, the results of these two experiments were combined, and *A. lancea* were redivided into three chemical types: Dabie Mountain Cangzhu (located in Dabie Mountain), Hubei-Anhui Cangzhu (including areas outside Dabie Mountain of Hubei, Anhui), and Henan Songshan Cangzhu (Takeda, 1994; Takeda et al., 1995; Takeda et al., 1996a; Takeda et al., 1996b).

In the present study, the analysis of the four main essential oil components revealed a continuous variation from south to north. Two main chemotypes of *A. lancea* essential oil were found in China. The results of cluster analyses of *A. lancea* populations based on the four main essential oil components indicated that 13 A*. lancea* populations were grouped into two main clusters. The *A. lancea* populations that are mainly distributed in Hubei, Anhui, Shaanxi, and the western part of Henan province were categorized as HBA, which has been characterized by a higher content of total essential oil with β-eudesmol and hinesol as the main components and atractylodin and atractylon as minor components. The other group was MA, distributed in Jiangsu, Shandong, Shanxi, Hebei, Inner Mongolia, and other northern regions. This chemotype has higher proportions of atractylodin and atractylon. Similar results were also found in the classification of the VOC metabolites. These results align with the division of *A. lancea* chemotypes based on the four main essential oil components.

In recent years, Dabieshan-type *A. lancea* has attracted the attention of researchers. This type mainly contains hinesol and β-eudesmol (Zhang et al., 2011; Lu et al., 2020). In this study, the Dabieshan-type *A. lancea* was classified into the HBA group, which includes the *A. lancea* production areas in Hubei, Anhui, and southern Henan province. Moreover, the *A. lancea* population from SXWZ displayed a distinctive chemical type of essential oil, with a higher proportion of hinesol and β-eudesmol, although the total content of essential oil was relatively low. Thus, further exploration of *A. lancea* from SXWZ is is warranted to better understand its unique characteristics.

### 4.2 Metabolic profiling of VOCs in *A. lancea* from different distribution areas

Numerous reports have documented VOCs as the primary medicinal constituents and aromatic substances of essential oils from rhizomes of *A. lancea* (Li et al., 2018; Navarro-Meléndez and Heil, 2014). Previous studies have shown that terpenoids and heterocyclic compounds are the major bioactive components of ALR. These chemicals are responsible for the various beneficial effects observed in extracts, such as anti-hypoxia, hypoglycemic, anti-ulcer, anti-virus and hepatoprotective activities (Ouyang et al., 2012; Xu et al., 2016). For instance, atractylone, which accounts for 20% of the sesquiterpenoids, has been reported to inhibit the growth of HepG2 cells by inducing apoptosis (Gu et al., 2019). Hinesol, comprising 5 to 9% of ALR, exhibits a strong inhibitory activity against *Helicobacter pylori* and potent antitrypanosomal activities *in vitro* (Otoguro et al., 2011).

Atractylodin, a heterocyclic compound, is also a major active ingredient of ALR. Numerous pharmacological experiments, both *in vivo* and *in vitro*, have demonstrated that atractylodin possesses several activities, including anti-inflammatory, anti-tumor and liver protective effects (Tang et al., 2018; Chuang et al., 2019; Chang et al., 2021). In addition, our present study also identified ketones and esters. Modern pharmacological research indicates that esters exhibit significant anti-inflammatory, blood pressure-lowering and stress-relieving activities, while ketones are strong antioxidants capable of eliminating free radicals (Taheri et al., 2020; Xu et al., 2021). The most common compounds among the 1427 VOCs identified in our study were aromatic hydrocarbons. This expands our knowledge of the volatile metabolic profiling of *A. lancea* and its potential applications.

At the population level, *A. lancea* in Maoshan, known as the Daodi herb, exhibits a clearly differentiated phenotype in terms of its essential oil content. Terpenoids and phenylpropanoids are the major constituents of this oil, contributing to the characteristic aroma and activities of ALR. These compounds are crucial for the plant’s adaptation to ever-changing environmental conditions. Terpenoids are frequently used as phytoalexins, and their accumulation is influenced by biotic or abiotic stresses, such as high altitude, cold weather, drought and nutrient deficiencies. Our results show that environmental and climatic factors have a strong impact on the biosynthesis of the secondary metabolites in ALRs. The accumulation of terpenoids and phenylpropanoids in ALR was found to be particularly sensitive to ambient environmental changes. This aligns with previous studies showing variations in metabolic substances among different ecotypes of *Cistanche deserticola*, *Lycium barbarum*, and dried jujube (Sun et al., 2020; Wang et al., 2020; Shi et al., 2022).

Plants are natural experts in organic synthesis, being able to generate large numbers of specific metabolites with widely varying structures that help them adapt to variable survival challenges. Genetic changes and environmental factors are the main causes of this chemical diversity. The geographical location or origin of a plant has a strong influence on the chemical diversity of the specialized metabolites, and this influence is usually attributed to the environment and climate during seedling establishment and subsequent growth of the plant. For example, a study showed that transplanted *A. lancea* plants exhibit chemical profiles more similar to those of plants in the destination region rather than the originating region (He et al., 1993). Therefore, in order to better protect the precious medicinal plant resource of *A. lancea*, it will be necessary to conduct comprehensive in-depth investigations on the ecological environment in the Maoshan area.

### 4.3 Geographical discrimination and authentication of high-quality herbs

Numerous recent studies have highlighted the significant impact of geographical location on the chemical composition of various plants (Guo et al., 2008; Yang et al., 2018; Zhang et al., 2022). Si et al. studied geographical discrimination and authentication of Chinese garlic using multi-element, volatile compound and metabolomics profiling combined with chemometrics. The results demonstrated the potential of chemical profiling in combination with chemometrics as feasible approaches for the authenticating the geographical origin of plants. Similarly, Zhang et al. identified three compounds that could serve as marker compounds for the quality control of *Fagopyrum dibotrys* from different origins (Zhang et al., 2022). Comparable results were reported in the chemometric discrimination of the geographical origin of licorice in China using untargeted metabolomics, which revealed a number of significantly different metabolites among licorice samples (Lu et al., 2022).

In the case of Fuzi and Fupian production in Jiangyou, Sichuan and Hanzhong, Shannxi, the quality of the products can vary considerably, with Jiangyou Fuzi being considered the Dao-di herb. The unique aconite alkaloids of Jiangyou Fuzi can be used as chemical markers for geographical discrimination and authentication of high-quality herbs. Lv et al. also identified quercetin and succinic acid as biomarkers for identification of Zhongning Goji berries using a nontargeted metabolomics approach (Lv et al., 2020). In the present study, the specific proportion of atractylodin and atractylon in Mao*-A. lancea* correlated with high quality, making these compounds as chemical markers for origin identification. Because the MA and HBA chemotypes identified in our study correlate with the levels of the four essential oils, categorization into one of these chemotypes can be used for quality control and standardization of *A. lancea* products in traditional Chinese medicine. Higher levels of atractylon and atractylodin are known to be characteristic of high-quality *A. lancea* (Wang et al., 2012; Zhang W. J. et al., 2021; Xu et al., 2022), and the HBA chemotype, which is mainly produced in Hubei, Anhui, Shaanxi, and a region west of Henan province, has lower atractylon and atractylodin but higher β-eudesmol and hinesol; therefore, we predict that the HBA chemotype is an indicator of lower quality *A. lancea*. The other chemotype that we identified was MA, which is characterized by atractylodin and atractylon as main components with lower amounts of hinesol and other components, making this chemotype more closely associated with high quality product. Accordingly, we found that the distribution area of the MA chemotype matches areas known to produce high-quality *A. lancea*, including Maoshan in Jiangsu Province, and Shandong, Shanxi, Hebei, Inner Mongolia, and other northern regions. In this study, we also identified 6 reliable compounds that can be used as chemical markers for the authentication of *A. lancea* arising from the three major regions (STA, NTA, and MSA) in which this plant is found. The concentration of all 6 chemical markers was significantly higher in the MSA group, followed by that of NTA and STA. MSA belongs to traditional Dao-di areas, representing high quality; consequently, the differentiation of chemotypes and the authentication of geographical origin can contribute to ensuring product efficacy and safety.

Metabolomics analysis provides a new perspective to understand the metabolic variations in traditional herbs grown in different geographical environments, thereby contributing to the identification of high-quality herbs (Chen et al., 2023). The two chemical types and three metabolic profiles identified in this study exhibited a strong correlation with geographical distribution, and our results showed that the volatile oil content of ALR varies continuously among different geographical populations. Therefore, targeted metabolomic analysis combined with quantitative chemical assays are worth developing to serve as authentication methods for *A. lancea* based on the chemotype profiles. Importantly, the two methods of detection that we developed can be mutually validated, further improving the reliability of research conclusions. However, the accurate authentication and traceability of plant geographical origin are still challenging. The current plant metabolomics technologies have certain limitations in achieving comprehensive qualitative and quantitative analysis of metabolites in all metabolic pathways of plants, and different analytical methods may lead to different results. In addition, these metabolomics data may not necessarily yield accurate results, as some non-experimental factors such as changes in ambient temperature, climate, soil, and other ecological environments can affect the proportions of plant metabolites. Therefore, it must be noted that with continuous improvement of isolation and detection techniques, the integrated application of multi-platform data and the development of bioinformatics, metabolomics will likely play an essential role in geographical discrimination and authentication of high-quality herbs.

## 5 Conclusion

The objective of this study was to determine the chemotypes of *A. lancea* across different distribution areas and identify chemical markers through the quantification of VOCs using a GC–MS/MS-based targeted metabolomics approach. The investigation revealed a gradual decrease in the content of four major essential oil components from south to north, indicating a latitudinal variation. Two main chemotypes of *A. lancea* essential oil were found in China. The first chemotype, termed HBA, was predominantly distributed in Hubei, Anhui, Shaanxi, and a region west of Henan province. It exhibited a higher content of total essential oil with β-eudesmol and hinesol as the main components, accompanied by minor components such as atractylodin and atractylon. The second chemotype, MA, encompassed plants from Jiangsu, Shandong, Shanxi, Hebei, Inner Mongolia, and other northern regions, and it exhibited higher concentrations of atractylodin and atractylon. Notably, *A. lancea* from SXWZ has distinctive essential oil components warranting further exploration. The comprehensive analysis here detected and identified 15 categories of VOC metabolites with their concentrations showing significant variations among samples from different distribution areas. Significant differences were observed in the profiles of terpenoid, heterocyclic compounds, esters, and ketones across different distribution areas. 6 compounds and 455 metabolites could be used as candidate markers for the authentication of *A. lancea* among the three major regions (STA, NTA, and MSA). In summary, the 6 reliable chemical markers identified were ketobemidone, 2,2’-bis(4,5-dimethylimidazole), 1,3-pentanedione, 4,4-dimethyl-1-phenyl-, (E, E, E)-2,6,9,9-tetramethyl-2,6,10-cycloundecatrien-1-one, β-santalol, and cembrene. The findings highlight the potential of metabolite profiling combined with chemometrics as viable approaches for the authentication of the geographical origin of *A. lancea*.

## Data availability statement

The original contributions presented in the study are included in the article/**Supplementary Material**. Further inquiries can be directed to the corresponding authors.

## Author contributions

CZ: Investigation, data analysis, and writing of original draft. HW: Writing and revision. SW and LG: Conception and design. YHW, JS, and YZ: Sample collection. CZ, ZX, and XG: Sample preparation and measurement. YFW: Data curation. MQ and CL: Data curation, formal analysis. LG: Methodology, funding acquisition. All authors contributed to the article and approved the submitted version.

## Supplementary material

Supplementary Figure 1 The morphological differences of *A. lancea* from distribution areas.

Supplementary Figure 2 A sample of *A. lancea* wild plant.

Supplementary Figure 3 The stacking diagram of total ion current (TIC) maps from mass spectra of quality control (QC) samples.

Supplementary Figure 4 The original three group classification of volatile metabolome data from 133 production areas based on *k*-means analysis.

Supplementary Figure 5 PLS-DA model analysis for the *A. lancea* samples from different distribution areas (R^2^X = 0.956, R^2^Y = 0.939 and Q^2 =^ 0.907).
