# Supplementary figures and images for: Authenticating the geographic origins of Atractylodes lancea rhizome chemotypes in China through metabolite marker identification
Source: Front Plant Sci. 2023 Sep 28;14:1237800. doi: 10.3389/fpls.2023.1237800 (PMC10569125; doi:10.3389/fpls.2023.1237800)

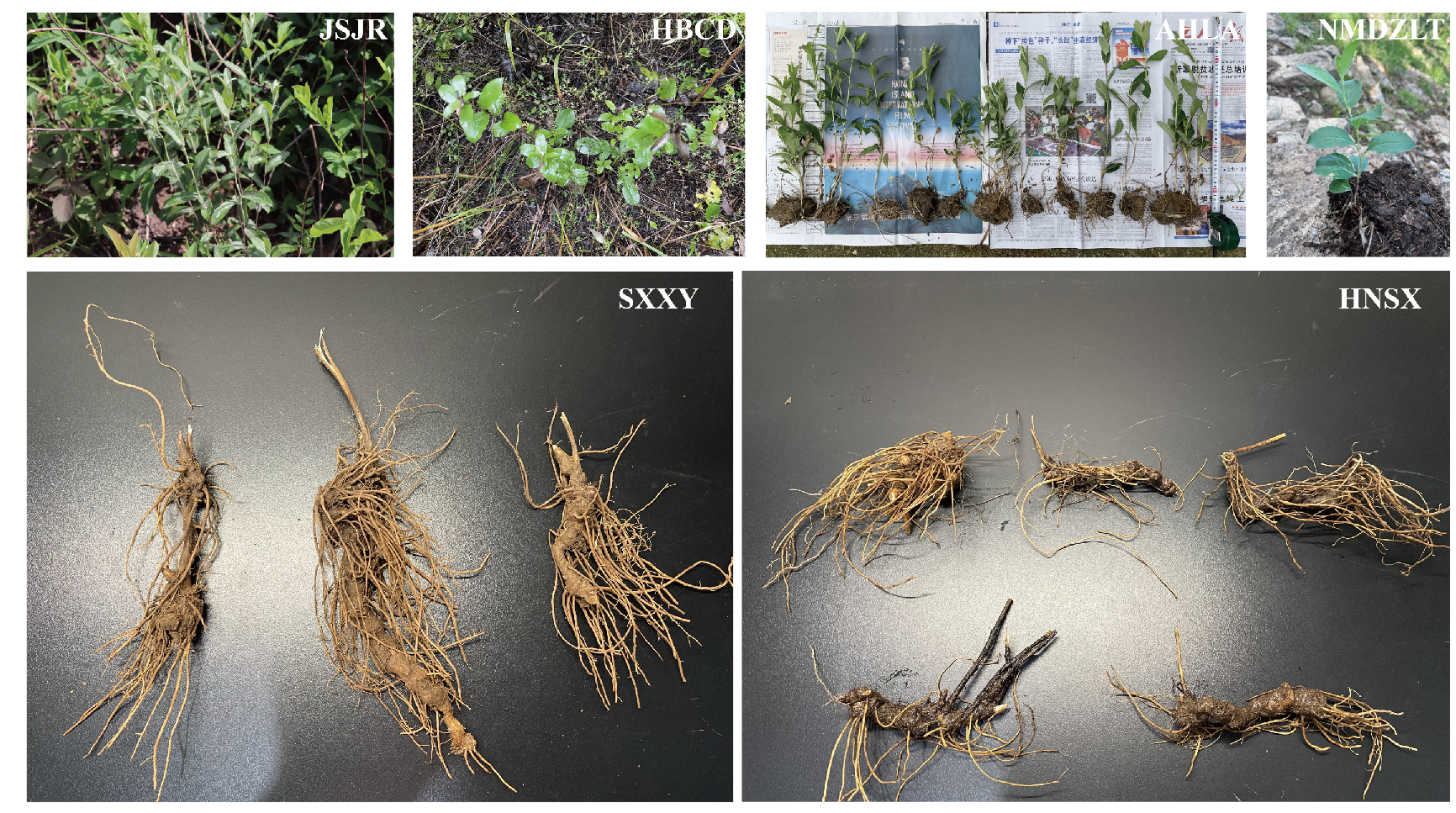

Supplement: Supplementary Figure 1 — The morphological differences of A. lancea from distribution areas. [file Image_1.tif]

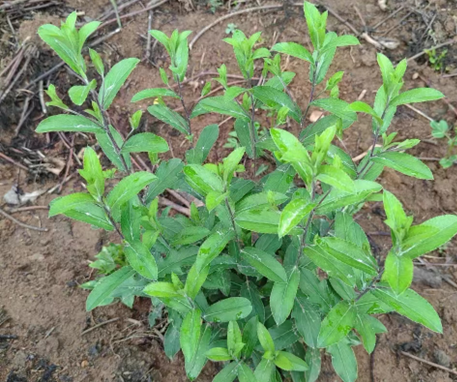

Supplement: Supplementary Figure 2 — A sample of A. lancea wild plant. [file Image_2.tif]

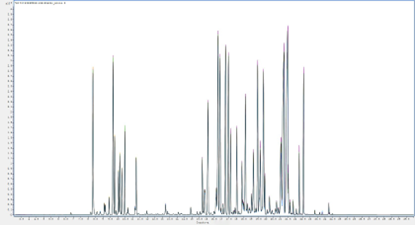

Supplement: Supplementary Figure 3 — The stacking diagram of total ion current (TIC) maps from mass spectra of quality control (QC) samples. [file Image_3.png]

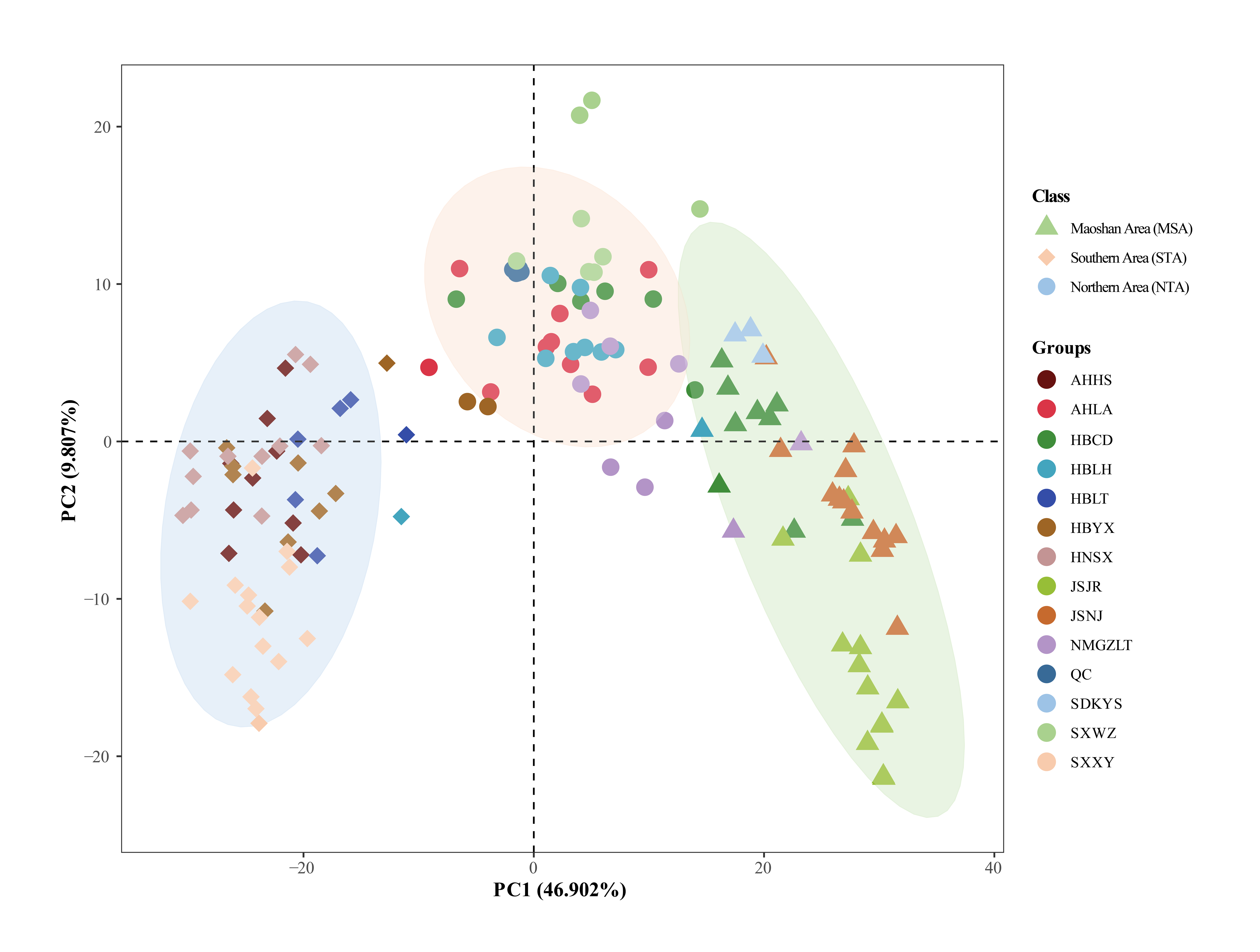

Supplement: Supplementary Figure 4 — The original three group classification of volatile metabolome data from 133 production areas based on k-means analysis. [file Image_4.tif]

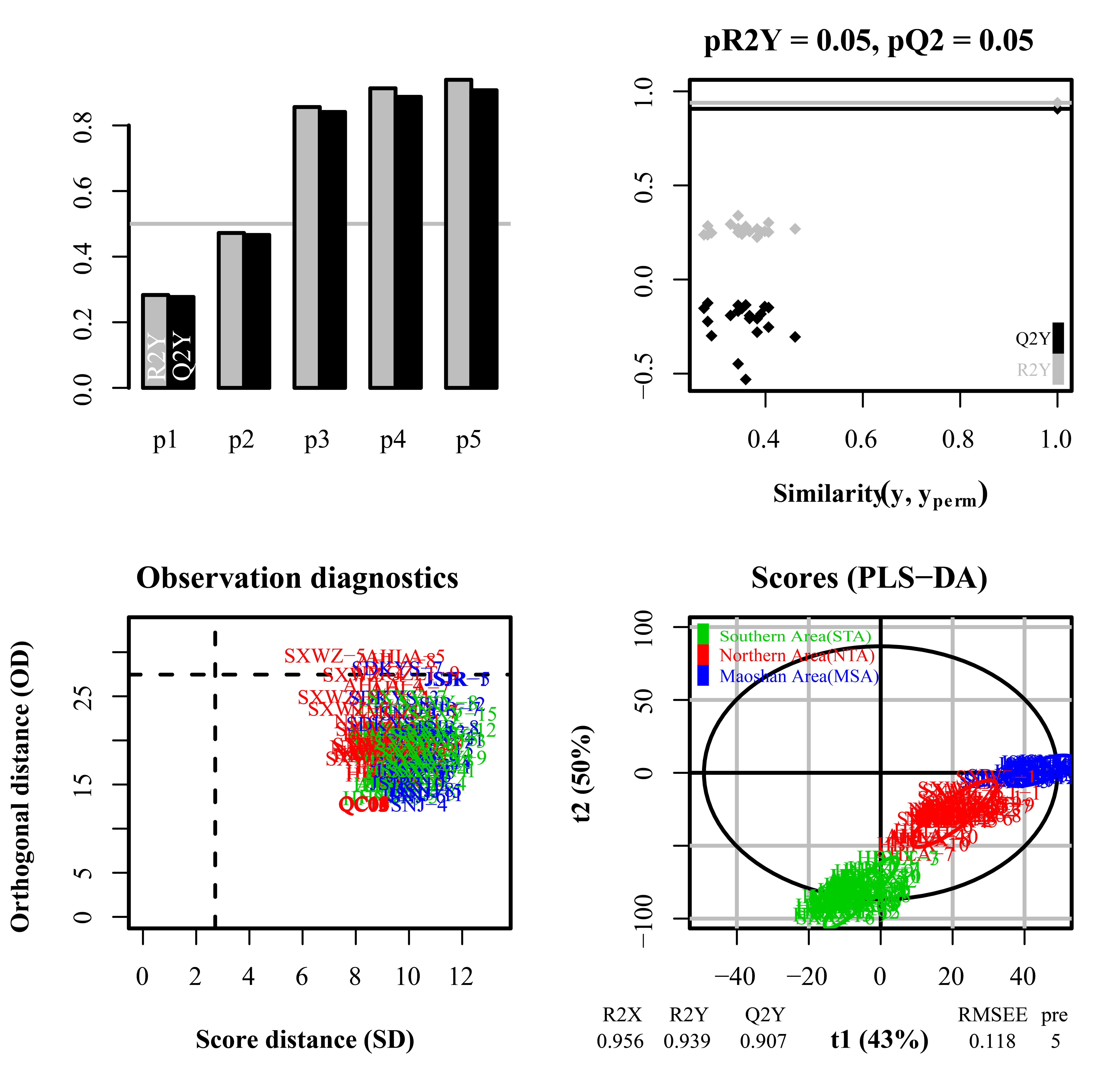

Supplement: Supplementary Figure 5 — PLS-DA model analysis for the A. lancea samples from different distribution areas (R2X = 0.956, R2Y = 0.939 and Q2 = 0.907). [file Image_5.tif]
